# Ambient Vibration Testing for Story Stiffness Estimation of a Heritage Timber Building

**DOI:** 10.1155/2013/198483

**Published:** 2013-10-08

**Authors:** Kyung-Won Min, Junhee Kim, Sung-Ah Park, Chan-Soo Park

**Affiliations:** Department of Architectural Engineering, Dankook University, Yongin 448-701, Republic of Korea

## Abstract

This paper investigates dynamic characteristics of a historic wooden structure by ambient vibration testing, presenting a novel estimation methodology of story stiffness for the purpose of vibration-based structural health monitoring. As for the ambient vibration testing, measured structural responses are analyzed by two output-only system identification methods (i.e., frequency domain decomposition and stochastic subspace identification) to estimate modal parameters. The proposed methodology of story stiffness is estimation based on an eigenvalue problem derived from a vibratory rigid body model. Using the identified natural frequencies, the eigenvalue problem is efficiently solved and uniquely yields story stiffness. It is noteworthy that application of the proposed methodology is not necessarily confined to the wooden structure exampled in the paper.

## 1. Introduction

Vibration-based structural health monitoring (SHM) is based on vibration testing of structures which requires high capacity actuators. However, exciting structures in a controlled and repeatable manner is rather limited in practice. In addition, the forced vibration testing is not preferred for heritage building structures where artificial loading might induce significant damage to the tested structures. Thus, monitoring of structural responses from ambient vibration is preferred, since dynamic properties can be identified by analyzing ambient responses of the buildings, for example, heritage court building [1], bell tower of the Monza's Cathedral [2], three representative monuments in Rome [3] (i.e., the Colosseum, Trajan's Column, and Aurelian Walls), and the historic Morca suspension footbridge [4]. All of these studies are exclusively for masonry or stony structures. 

In Northeast Asia (i.e., Korea, China, Japan, etc.), timber buildings had been traditionally constructed for majestic superstructures on gates of stony castle walls and then have been preserved as heritages so far. These days, portions of the heritage buildings are exposed to rather strong ambient vibration sources. Excitation induced by passage of traffics propagates to the structures. The repeated support excitation might induce functional problems to sensitive equipments or structural damage at the structures. Structural vibration might trigger damage especially for the structures with weathered structural members and weakened structural integrity. In the context, analytical and experimental studies on structural behaviors of historic timber buildings have been conducted: analytical procedure of finite element method was applied to ancient Chinese timber architecture [5]; shaking table and static tests were carried out with scaled models of Japanese temples [6]. 

Structural vibrations derived from support excitations can be used to characterize the structure [7]. The structural vibratory responses depend on both spectral content of the excitations and dynamic characteristics of the structure. Assuming that the support excitations are random, structural responses represent directly the structural dynamic characteristics which are useful to understand behavior of the structure and further assess structural integrity. For example, natural frequencies and mode shapes can be used for examining overall structural stiffness. The structural stiffness is important especially for the heritage timber structures. At the typical timber framing, all connections between columns and beams are neither rigid nor pinned, since the structural members are fitted with joints secured by wooden pegs. Therefore, estimation of structural stiffness of the timber structures has been considered challenging so far.

The Dongdaemun, designated as the Korean treasure no. 1, is a historic timber structure: it is a two-story wooden building on the platform of stone foundations. It is located at an intersection of busy roads in downtown Seoul being exposed to traffic vibrations. In this study, ambient vibration testing is conducted for the Dongdaemun for the purpose of story stiffness evaluation. The structural responses of ambient vibrations are measured and then analyzed by output-only system identification for estimation of the modal parameters. A simplified rigid body model is adopted to efficiently model the Dongdaemun based on the identified mode shapes. Then, using the identified natural frequencies, an eigenvalue problem of the rigid body model is solved for estimating story stiffnesses of the structure. 

## 2. Ambient Vibration Testing of a Historic Structure 

### 2.1. The Dongdaemun, Korea

**Figure 1:**
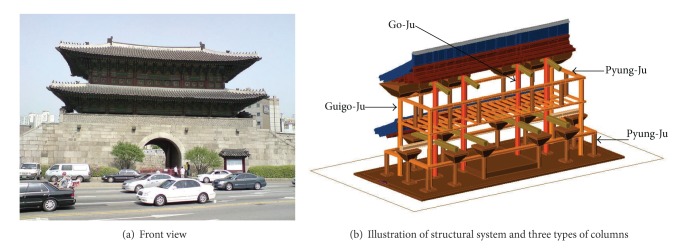
The Dongdaemun (great gate in the East).

Castle walls surrounding Hanyang (the old capital of Korea, now the downtown area of Seoul) were built in Joseon (the final ruling dynasty of Korea, lasted from 1392 to 1910). Among eight gates along the castle walls, the one located at the East is the Dongdaemun, which stands for great gate in the East. The Dongdaemun was originally constructed in 1396, repaired in 1453, and finally rebuilt in 1869. It has two types of distinct structures, as shown in Figure 1(a). The upper structure is a timber frame consisting of beams and supporting columns (i.e., postings), and the lower structure is a massive platform foundation made with stones. The columns and beams of the two-story wooden structure are fitted with joints secured by mortise and tenon without metallic fasteners supporting eaves, bracket complexes and roofs, and so forth. 

**Figure 2:**
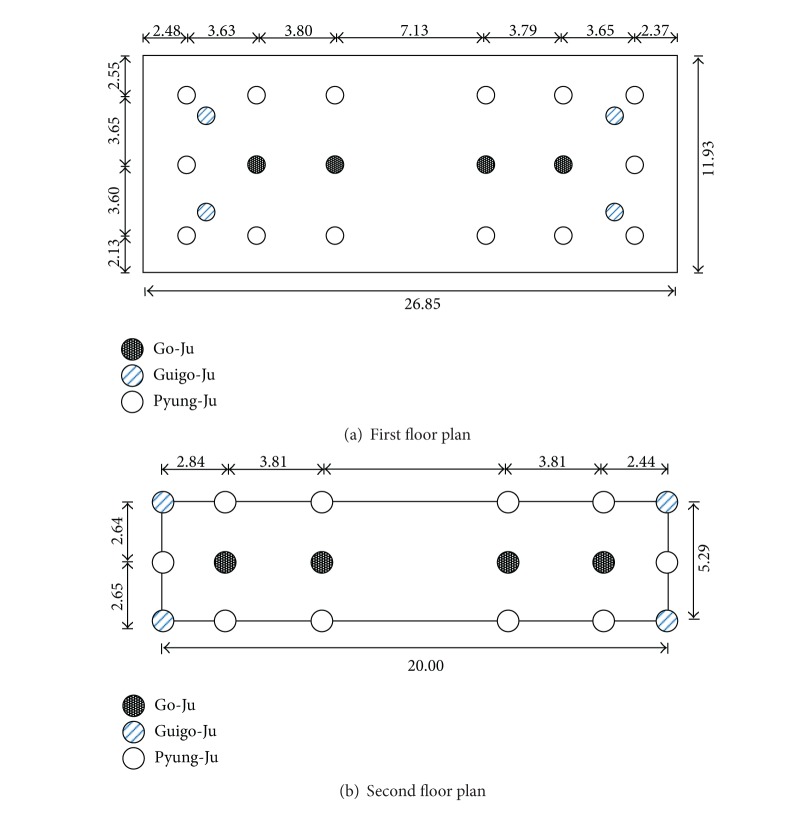
Layout of columns (unit: m).

Three types of columns (Figure 1(b)) support the structure: (1) four core columns (termed Go-Ju) are located along the center line in a row connecting foundations and roof beams; (2) four corner columns (termed Goigo-Ju) are located at four edges of the structure connecting foundations and the second floor; (3) numerous interstory columns (termed Pyung-Ju) are built up along the four sides at each floor delivering dead load to the floor beams. The three types of columns are also illustrated at each floor plan in Figure 2. Because of the rectangular footprint of the structure, it has two directions of motion: a flexurally strong axis along the long side of the rectangle is referred to as the longitudinal direction in the paper; the orthogonal direction is a flexurally weak axis and referred to as the lateral direction.

### 2.2. Ambient Vibration Testing

The Dongdaemun is located at an intersection of roads and above two subway lines. Excitation by ambient vibration sources (i.e., passages of vehicles and trains) is conveniently utilized as input to the structure. However, monitoring of structural responses with a high density of sensor array is necessitated due to the unique characteristics of timber frame and complex structural system of the Dongdaemun. To provide a high nodal density using a limited number of sensor nodes, reconfiguration of sensor installation is selected in the ambient vibration testing.

**Figure 3:**
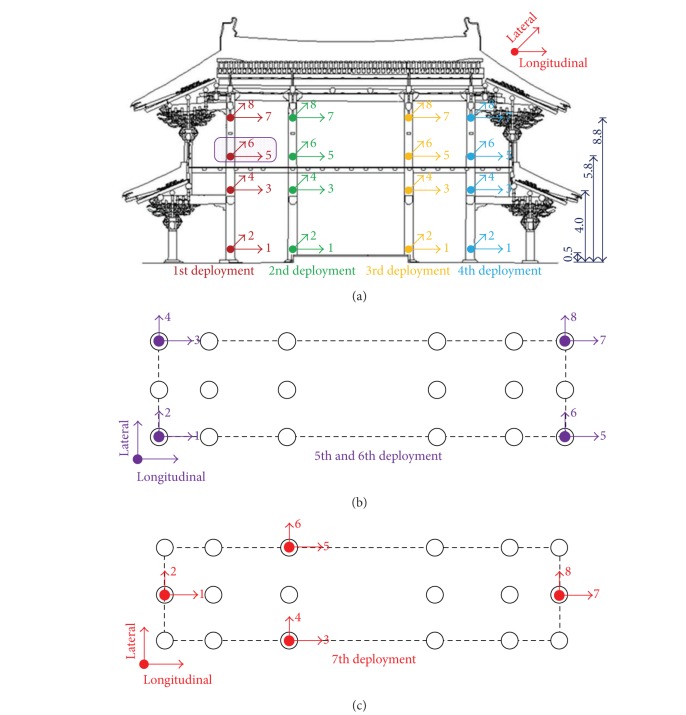
Schematics of multiple deployments of accelerometers. (a) Four sensor deployments at Go-Ju's with elevations noted (unit: m): the fixed reference sensor pairs are highlighted. (b) Two sensor deployments at Guigo-Ju's (elevations of 5.8 and 8.8 m). (c) A sensor deployment at Pyung-Ju's (elevation of 8.8 m).

A reconfiguring strategy with up to ten sensor nodes is adopted with the system redeployed six times after the initial deployment with two nodes overlapped. Pairs of accelerometers deployed along longitudinal and lateral directions are installed at each setup. For example, four pairs of sensors are installed at the first Go-Ju (Figure 3(a)) with the elevations of 0.5, 4.0, 5.8, and 8.8 m. Once the installation is complete, monitoring of ambient vibration is conducted. Then, sensors except the nodes 5 and 6 at the first Go-Ju highlighted are reinstalled at another Go-Ju, and two more sensors are installed at the nodes 5 and 6 at the Go-Ju. Similarly, testing and reinstallation are sequentially conducted twice for the remaining Go-Ju's. After completion of ambient vibration testing at Go-Ju's, the four pairs of moving sensors are installed twice at four Guigo-Ju's with the elevations of 5.8, and 8.8 m, respectively for each setup (Figure 3(b)). Finally, ambient vibration testing at Pung-Ju's is once conducted with sensor installation at the elevation of 8.8 m (Figure 3(c)). It should be noted that sensors at nodes 5 and 6 at the first Go-Ju collected data during the ambient vibration testing at Guigo-Ju's and Pyung-Ju's. This reconfiguring strategy realized a dense nodal configuration with 10 sensors installed in 48 nodes at the tested structure.

For measurement of weak ambient vibration in the seven tests, a multichannel high resolution data acquisition system interfaced by accelerometers with high sensitivity is adopted: a National Instruments 16-bit data acquisition system (NI USB-6210) is used with PCB Piezotronics 393B12 integrated circuit piezoelectric (ICP) accelerometers interfaced. The accelerometer measurement range is ±0.5 g, and its spectral noise floor of 100 Hz bandwidth is 0.07 *μ*g/*√*Hz. The accelerometer is well suited for civil structural monitoring because of its high sensitivity (10 V/g). The data is collected for 480 sec using a 30 Hz sampling frequency.

## 3. Output-Only Modal Analysis

### 3.1. Output-Only Modal Analysis Theory Revisited

Output-only modal parameter estimation techniques are categorized into two distinct groups depending on their analysis domains: frequency domain methods dealing with output spectra or power spectral density (PSD) and time domain methods using correlation (i.e., projection in subspace) of past and future outputs. In this study, a frequency domain technique called frequency domain decomposition (FDD) [1] is adopted to estimate the modal parameters of the Dongdaemun. The FDD is basically an output-only version of the conventional complex mode indicator function (CMIF) method [8]. PSD relationship between the system input, **u**, and the measured output, **y**, is expressed in continuous-time frequency domain as follows:

(1) $$\begin{array}{c} G_{yy}\left(j\omega \right)=H\left(j\omega \right)G_{uu}\left(j\omega \right)H^{H}\left(j\omega \right), \end{array}$$

where **G**_**uu**_(*jω*) is the PSD matrix of the input; **G**_**yy**_(*jω*) is the PSD matrix of the output; **H**(*jω*) is frequency response function (FRF) matrix; **H** ^*H*^(*jω*) is its complex transpose conjugate. If the system input is assumed as white noise, **G**_**uu**_(*jω*) will simply be a constant matrix in frequency axis; hence, **G**_**yy**_(*jω*) is directly proportional to the product of FRFs, **H**(*jω*)**H** ^*H*^(*jω*). By applying singular value decomposition (SVD) to (1), the output PSD matrix can be decomposed into singular vectors (mode shapes) and singular values of dominant frequencies. 

As a counterpart of the frequency domain method of FDD, this study also adopts the stochastic subspace identification (SSI) [8, 9] as a time domain alternative. The output block Hankel matrix is constructed from the measured 2*i* + *j* − 1 structural output vector sequence and partitioned as past output and future output as follows:

(2) Y0 ∣ 2i−1=[y0y1⋯yj−1⋮⋮⋱⋮yi−1yi⋯yi+j−2yiyi+1⋯yi+j−1⋮⋮⋱⋮y2i−1y2i⋯y2i+j−2]=[Y0 ∣ i−1Yi ∣ 2i−1]=[YpYf].

The main role of the partitioned output block Hankel matrix is preparing orthogonal projection where the past output works as instrumental variables for elimination of bias estimates due to colored noise output [10]. Two orthogonal projections of the row space of the future output, **Y**_*f*_, on the row space of the past output,  **Y**_*p*_, can be determined through LQ decomposition of the output block Hankel matrix:

(3) $$\begin{array}{c} P_{i}∶=\frac{Y_{f}}{Y_{p}};\;\;P_{i-1}∶=\frac{Y_{f}^{-}}{Y_{p}^{-}}, \end{array}$$

where **Y**_*f*_^−^ and **Y**_*p*_^−^ are defined as a one block row downshift in (2) as **Y**_0∣*i*_ and **Y**_*i*+1∣2*i*−1_, respectively. SVD is applied to factorize the projection **P**_*i*_:

(4) $$\begin{array}{c} P_{i}={USV}^{T}≅\left[\begin{array}{cc} U_{1} & U_{2} \end{array}\right]\left[\begin{array}{cc} S_{1} & 0\\ 0 & 0 \end{array}\right]\left[\begin{array}{c} V_{1}^{T}\\ V_{2}^{T} \end{array}\right]=U_{1}S_{1}V_{1}^{T}. \end{array}$$

Since the projection is equal to the product of the extended observability matrix and the nonstationary Kalman filter state sequence [9], the extended observability matrix and the nonstationary Kalman filter state sequence are calculated respectively as

(5) $$\begin{array}{c} {𝒪}_{i}=U_{1}S_{1}^{1/2};\;\;{\hat{X}}_{i}=S_{1}^{1/2}V_{1}^{T}. \end{array}$$

The one-step shifted state sequence is also calculated as

(6) $$\begin{array}{c} {\hat{X}}_{i+1}={\left({𝒪}_{i-1}\right)}^{†}P_{i-1}, \end{array}$$

where *𝒪*_*i*−1_ is equivalent to *𝒪*_*i*_ with the last block row omitted. Furthermore, † is the pseudoinverse operator. Finally, estimates of system matrices of **A** and **C** are calculated by a least-squares solution:

(7) $$\begin{array}{c} \left[\begin{array}{c} \hat{A}\\ \hat{C} \end{array}\right]=\left[\begin{array}{c} {\hat{X}}_{i+1}\\ Y_{i\;|\;i} \end{array}\right]{\hat{X}}_{i}^{{\;}^{†}}. \end{array}$$

Modal parameters can be estimated from the estimated system matrices. The estimated system matrix $\hat{A}$ can be decomposed by eigen decomposition as $\hat{A}={\Psi \Lambda \Psi }^{-1}$, where diagonal matrix Λ = diag⁡(*λ*_*di*_) consists of the discrete-time complex eigenvalues. Ψ contains eigenvectors in each column. The discrete-time eigenvalues are first converted to continuous-time eigenvalues *λ*_*ci*_ as *λ*_*ci*_ = ln⁡(*λ*_*di*_)/Δ*t*, where Δ*t* is the time step of the digital data acquisition system. The natural frequencies *ω*_*ni*_ and damping ratios *ς*_*i*_ can then be easily calculated from the conjugate pair of complex-valued eigenvalues: $\lambda_{ci},\lambda_{ci}^{*}=-\varsigma_{i}\omega_{ni}\pm j\omega_{ni}\sqrt{1-\varsigma_{i}^{2}}$. The mode shape vector for the *i*th mode Φ_*i*_ can be calculated as $\left[\begin{array}{ccccc} \Phi_{1} & \cdots & \Phi_{i} & \cdots & \Phi_{n} \end{array}\right]=\hat{C}\Psi$.

### 3.2. Modal Parameter Estimation

The measured acceleration data are analyzed to identify the modal characteristics of the Dongdaemun without known input loading. The quality of the estimated modal parameters by the FDD method is governed by the estimated PSD functions. The PSD function calculated for each sensor location is improved by using a Hanning window on the time-history data prior to the use of the fast Fourier transform (FFT) algorithm. In addition, repeated Fourier spectra calculated from time-history records with 50% overlap between them in the time domain are averaged. This approach to improving the PSD spectra provides a good tradeoff between the reduction of noise and the distinctive qualities of the modal peaks [11].

**Figure 4:**
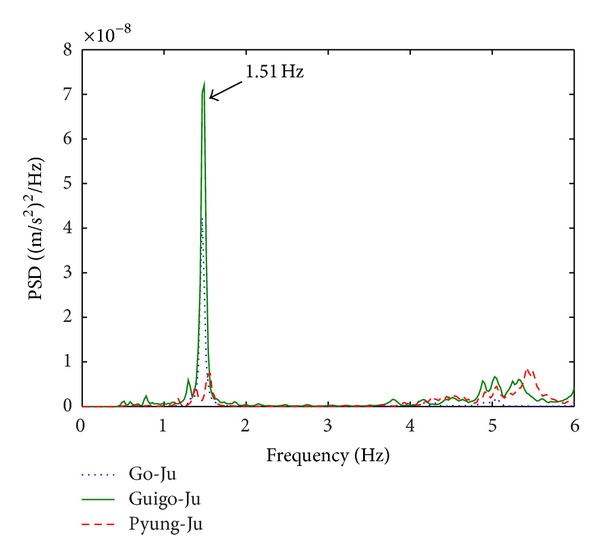
PSD spectra in longitudinal direction.

**Figure 5:**
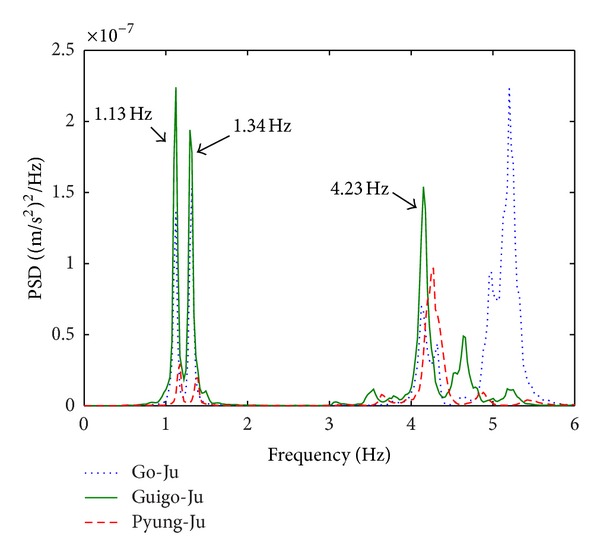
PSD spectra in lateral direction.

Figure 4 presents the PSD spectra of the measured longitudinal accelerations at sensor nodes 5, 1, and 1, respectively, for the 1st, 6th, and 7th sensor deployments as representatives of Go-Ju, Guigo-Ju, and Pyung-Ju. A dominant frequency of 1.51 Hz is observed at the three cases. The PSD spectra of the measured lateral accelerations at the sensor nodes 6, 2, and 2, respectively, for the 1st, 6th, and 7th sensor deployments are given in Figure 5. Sharp peaks at 1.13, 1.34, and 4.23 Hz seen at the three cases imply modal frequencies in lateral direction revealing complex vibratory behavior of the structure in the lateral direction.

Two output-only system identifications are conducted by the frequency-domain FDD and time-domain SSI with the collected ambient vibration data. As for the FDD method, by selecting dominant peaks in the previously calculated PSDs of Figures 4 and 5, natural frequencies are determined, and mode shapes are automatically acquired from the corresponding first singular vector. For the purpose of damping estimation, a variation of half power method, so-called enhanced FDD [12], is adopted using the PSD spectra in lieu of frequency response function. The time-domain SSI is independently conducted with the identical data, and modal parameters are determined. Results of the estimated modal parameters by FDD and SSI are tabulated in Table 1.

Table 1 shows natural frequencies and damping ratios in the lateral and longitudinal directions. Overall, the natural frequencies identified by the two methods are very close. However, the damping ratios are a little different, which implies the uncertainty of damping estimation from output-only system identification in the literature [13]. Damping ratios in the lateral direction are larger than those in the longitudinal direction. As a result, flexible and deformable behavior is expected in lateral direction.

**Figure 6:**
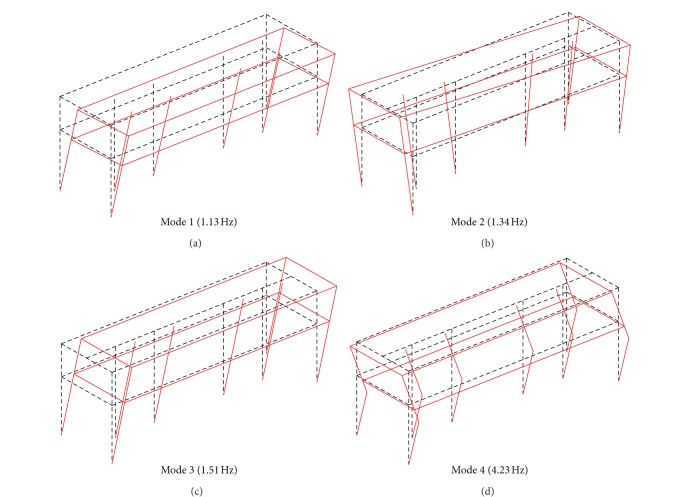
FDD derived mode shapes.

The mode shapes calculated for each subsection of the structure are stitched together using the collocated sensors to yield the full global modes of the structure respectively for the two FDD and SSI methods. For quantitative comparison, the modal assurance criterion (MAC) [14] is adopted in this study. The MAC is a scholar index to correlate two sets of mode vectors, defined as

(8) $$\begin{array}{c} \text{MA}{\text{C}}_{i}\left(\Phi_{\text{FDD},i}^{}\cdot \Phi_{\text{SSI},i}^{}\right)=\frac{{\left(\Phi_{\text{FDD},i}^{T}\cdot \Phi_{\text{SSI},i}^{}\right)}^{2}}{\left(\Phi_{\text{FDD},i}^{T}\cdot \Phi_{\text{FDD},i}^{}\right)\left(\Phi_{\text{SSI},i}^{T}\cdot \Phi_{\text{SSI},i}^{}\right)}, \end{array}$$

where Φ_*A*,*i*_ is the *i*th mode from the method *A*. The MAC ranges from 0 to 1: the value of 1 implies perfect correlation of two mode shape vectors, while the value close to 0 means the uncorrelated vectors. In general, it is accepted that the MAC greater than 0.80 to 0.85 is considered a good match [4]. The MACs calculated with the identified mode shapes in this study are listed in Table 2: the lowest MAC of 0.9855 implies an excellent match between the mode shapes from the two methods. Since the two sets of mode shapes are very close, the mode shapes derived from the FDD are presented in Figure 6, where original shapes of the structure are superimposed by dotted lines. As seen, very clear mode shapes which are noticeable amplitudes in the directions of motion are almost identical at all nodes. Thus, it can be concluded that the structure behaves as an integrated body in the vibratory motion.

## 4. Model-Based Story Stiffness Estimation

### 4.1. Simplified Rigid Body Model

Finite element model updating [15, 16] and data-driven structural parameter estimation [17, 18] can be applied to identify structures. However, these approaches are deemed to be rather challenging for its direct application to this study, since the tested structure is a complex timber frame with numerous structural elements. In this regard, a different identification approach of model based data fitting is sought in this study.

The estimated modal characteristics of the Dongdaemun aforementioned in Section 3.2 confirm that identical dominant frequencies are noticed in the PSD spectra regardless of sensor deployments and sensor nodes, which leads to the speculation that the structure behaves as an integrated body. Therefore, a simplified rigid body model with two lumped masses is suggested to idealize the structure under the assumption that sum of flexural rigidities of beam-columns can be replaced equivalently with story stiffness at each mass. In the model, all connections between masses can be considered rotation-free hinges. 

**Figure 7:**
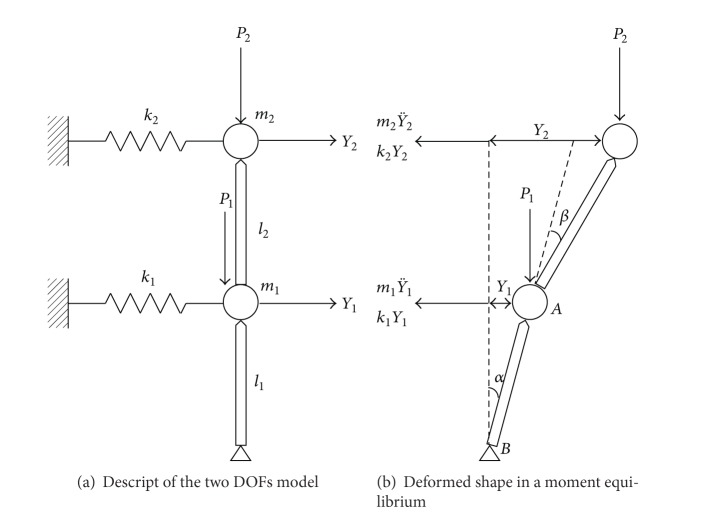
Simplified rigid body model.

As seen in Figure 7, two degrees of freedom are considered for translational motions at each floor. *m*_1_ and *m*_2_ present lumped masses, respectively, for the first and second floors; *k*_1_ and *k*_2_ are equivalent story stiffnesses, respectively, for the first and second floors; *P*_1_ and *P*_2_ are weights of each floor acting in the direction of gravity; inter-story heights are *l*_1_ and *l*_2_, respectively, for each floor; rigid body motions are described as displacements of *Y*_1_ and *Y*_2_, respectively, for each floor.

The equation of motion for the model can be derived using an instant deformed shape of the model with displacements *Y*_1_ and *Y*_2_, respectively, for the first and second stories shown in Figure 6(b). Considering dynamic moment equilibriums at points *A* and *B* and ignoring structural damping phenomena [19] leads to

(9) P2l2sin(α+β)=k2Y2  l2cos⁡(α+β)+m2Y¨2l2cos⁡(α+β),P2(l2sin(α+β)+l1sinβ)+P1l1sinβ  =(k2Y2+m2Y¨2)   ×(l2cos⁡⁡(α+β)+l1cos⁡β)   +(k1Y1+m1Y¨1)l1cos⁡β.

Assuming rotational angles *α* and *β* are small, the trigonometric functions in (9) can be simplified. Then, combining (9), a linear equation for dynamic motion of the model is written in matrix expression as

(10) [m100m2]{Y¨1Y¨2}   +[k1−P1l1−P2(l1+l2)l1  l2P2l2P2l2k2−P2l2]{Y1Y2}={00}.

Equation (10) can be further symbolized as

(11) $$\begin{array}{c} M\ddot{Y}+KY=0, \end{array}$$

which stands for an eigenvalue problem, provided that a harmonic motion is considered. Namely, the eigenvalue problem of (11) captures vibratory behavior of the Dongdaemun.

### 4.2. Story Stiffness Estimation

Structural integrity of the Dongdaemun is assessed by story stiffness estimation of the simplified rigid body model: the story stiffnesses are estimated using the vibration model derived as (10) and the modal parameters estimated from the ambient vibration testing. The other parameters in (10) should be known in advance based on geometrical and material properties regarding the structure; referring to the report on field examination of Dongdaemun issued by the authority [20], the parameters are given as *m*_1_ = 290 t, *m*_2_ = 315 t, *l*_1_ = 5.84 m, *l*_2_ = 7.69 m, *P*_1_ = 5,934 kN, and *P*_2_ = 3,082 kN. The eigenvalue problem of (11) can be easily solved by setting determinant of the matrix equation to zero:

(12) $$\begin{array}{c} \det\left|\left[\begin{array}{cc} k_{1}-\frac{P_{1}}{l_{1}}-\frac{P_{2}\left(l_{1}+l_{2}\right)}{l_{1}\;l_{2}} & \frac{P_{2}}{l_{2}}\\ \frac{P_{2}}{l_{2}} & k_{2}-\frac{P_{2}}{l_{2}} \end{array}\right]-\omega^{2}\left[\begin{array}{cc} m_{1} & 0\\ 0 & m_{2} \end{array}\right]\right|\;=0. \end{array}$$

Substituting the given parameters and natural frequencies identified previously into (12), story stiffnesses are uniquely calculated for each vibratory direction of the Dongdaemun. As a result, the story stiffnesses in the lateral direction are calculated as 15.1 and 15.2 kN/mm, respectively, for *k*_1_ and *k*_2_. In the longitudinal direction, 27.1 and 28.0 kN/mm are yielded, respectively, for *k*_1_ and *k*_2_. 

## 5. Conclusions

A Korean heritage property of the Dongdaemun is a historic wooden structure surrounded by lots of traffic such as subway trains, cars, and buses. In this study, a series of ambient vibration tests were conducted to identify the modal parameters of the structure. A dense sensor array was achieved by the virtue of multiple deployments of sensors sharing a small number of fixed reference sensors. Using time-history response data collected from the structure exposed to ambient excitation, offline output-only modal analysis was conducted by frequency domain decomposition and stochastic subspace identification methods. The four modes (i.e., two lateral, one longitudinal, and one torsional modes) were successfully identified. The modal parameters estimated from the two methods were also in a strong agreement. 

Based on the experimental findings, a simplified rigid body model was derived for story stiffness estimation. Using the identified natural frequencies and vibration equation described as eigenvalue problem, the story stiffness was calculated. Future work is focused on development of methodology to track structural deterioration for heritage structures based on the proposed story stiffness estimation; schedule-based monitoring of story stiffness of structures investigated gives a means for structural condition assessments along time line. 

## Figures and Tables

**Table 1:** Identified natural frequencies and damping ratios.

Modes	Descriptions	ω_FDD_ (Hz)	ω_SSI_ (Hz)	ς_FDD_ (%)	ς_SSI_ (%)
1	Transversal (1st lateral mode)	1.13	1.11	2.35	3.07
2	Torsional	1.34	1.35	2.04	3.50
3	Transversal (1st longitudinal mode)	1.51	1.51	1.38	2.00
4	Transversal (2nd lateral mode)	4.23	4.20	1.56	2.32

Note: The desciptions of each mode are illustrated in Figure 6.

**Table 2:** MACs for corresponding two sets of mode shapes.

Modes	1	2	3	4
MAC	0.9965	0.9855	0.9976	0.9940
